# Small Bragg-plane slope errors revealed in synthetic diamond crystals

**DOI:** 10.1107/S1600577520012746

**Published:** 2020-10-26

**Authors:** Paresh Pradhan, Michael Wojcik, Xianrong Huang, Elina Kasman, Lahsen Assoufid, Jayson Anton, Deming Shu, Sergey Terentyev, Vladimir Blank, Kwang-Je Kim, Yuri Shvyd’ko

**Affiliations:** a Advanced Photon Source, Argonne National Laboratory, Argonne, IL 60439, USA; b Technological Institute for Superhard and Novel Carbon Materials, 142190 Troitsk, Russian Federation

**Keywords:** diamond, crystals, X-ray Bragg diffraction, slope errors, silicon

## Abstract

Wavefront-preserving X-ray diamond crystal optics are essential for numerous applications in X-ray science. Perfect crystals with flat Bragg planes are a prerequisite for wavefront preservation in Bragg diffraction. However, this condition is difficult to realize in practice because of inevitable crystal imperfections. Here, X-ray rocking curve imaging is used to study the smallest achievable Bragg-plane slope errors in the best presently available synthetic diamond crystals and how they compare with those of perfect silicon crystals. It is shown that the smallest specific slope errors in the best diamond crystals are about 0.08 (3) µrad mm^−2^. These errors are only 50% larger than the 0.05 (2) µrad mm^−2^ specific slope errors measured in perfect silicon crystals. High-temperature annealing at 1450°C of almost flawless diamond crystals reduces the slope errors very close to those of silicon. Further investigations are in progress to establish the wavefront-preservation properties of these crystals.

## Abstract 2

X-ray rocking curve imaging is used to study the smallest achievable Bragg-plane slope errors in the best presently available synthetic diamond crystals and how they compare with those of perfect silicon crystals

## 1. Introduction   

Diamond features a unique combination of outstanding physical properties perfect for numerous X-ray crystal optics applications where traditional materials such as silicon fail to perform. Diamond is the material of choice in applications requiring improved transparency to X-rays, and the highest X-ray Bragg reflectivity, thermal conductivity, mechanical stiffness and resilience to radiation damage. Diamond optics are essential for tailoring X-rays to the most challenging requirements of X-ray research. Diamond optics are becoming vital for the generation of fully coherent hard X-rays by seeded X-ray free-electron lasers [see the recent review paper by Shvyd’ko *et al.* (2017 ▸) for details and references].

Progress in the fabrication of synthetic high-quality diamond crystals has been substantial in the last two decades. Crystals with defect-free areas of ∼4 mm × 4 mm and more, grown by a temperature-gradient method under high pressure and high temperature (HPHT), are now state of the art (Burns *et al.*, 2009 ▸; Polyakov *et al.*, 2011 ▸; Shvyd’ko *et al.*, 2011 ▸; Sumiya & Tamasaku, 2012 ▸; Stoupin *et al.*, 2016*a* ▸). However, the perfection of diamond crystals is typically not as high as that of silicon crystals, which are standard in X-ray crystal optics applications. In particular, the wavefront-preservation properties, critical for many applications, suffer from insufficient crystal quality.

Perfect crystals with flat Bragg planes are a prerequisite for wavefront preservation in Bragg diffraction. But nothing is perfect. How flat can Bragg crystal planes be in the best available diamond crystals? What are the smallest achievable Bragg-plane slope errors in the best presently available synthetic diamond crystals? How do these compare with those in perfect silicon crystals? These questions are addressed in the present paper.

In the studies presented here, Bragg-plane slope errors are measured using X-ray Bragg diffraction rocking curve imaging (RCI), also known as sequential topography (Lübbert *et al.*, 2000 ▸). This technique is applied to best-available diamond crystals featuring relatively large areas (∼4 mm × 4 mm) almost free of dislocations, stacking faults, inclusions and other defects detectable by white-beam X-ray topography (Tuomi *et al.*, 1974 ▸; Bowen & Tanner, 1998 ▸; Tran Thi *et al.*, 2017 ▸), which is used to prescreen the diamond crystals. The Bragg-plane slope errors in diamond crystals are compared with those in the highest quality reference silicon crystals.

RCI data also provide access to the specific dispersion 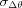 of the rocking curve widths Δθ. Normalized to the Bragg reflection width Δθ, it is a measure of the deviation from the largest Bragg reflectivity achievable by a perfect crystal.

The paper is organized as follows. In Section 2 we provide the results of RCI studies in a reference silicon crystal. The results of studies in selected freestanding diamond crystals and comparison with the reference silicon crystal are presented in Section 3. The design, fabrication and RCI studies of diamond crystals with strain-relief features mounted in crystal holders are discussed in Section 4. The effects of high-temperature heat treatment on the Bragg-plane slope errors in diamond crystals are discussed in Section 5. Supplementary data obtained with improved beamline X-ray optics are discussed in Section 6. We refer the reader to Appendix *A* [App appa] for details of the RCI technique and to Appendix *B* [App appb] for methods of mitigating the impact of beamline wavefront distortions on the actual values of the Bragg-plane slope errors. Appendix *C* [App appc] provides details of high-temperature annealing.

## 2. Bragg-plane slope errors in reference silicon crystal   

Prior to studying Bragg-plane slope errors in a diamond crystal, we used the same RCI technique and setup (introduced in Appendix *A* [App appa]) to measure RCI maps and the relevant crystal parameters in a specially prepared reference silicon crystal. These measurements were performed to establish a reference for the diamond crystals and to benchmark the ultimate performance of the RCI setup used in the later studies.

The reference crystal was manufactured from the highest quality high-resistivity single-crystal silicon, with the (531) crystal planes parallel to the surface. The 531 Bragg reflection is used to match the 531 Bragg reflection from the conditioning crystal. The crystal was made relatively large (15 mm × 15 mm × 15 mm). Its lower part was fixed in a crystal holder in a manner that did not create strain in the upper part, which was exposed to X-rays.

**Figure 1:**
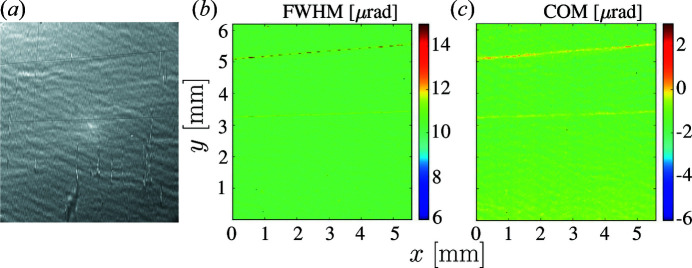
An X-ray image and rocking curve maps of a reference silicon crystal in the 531 Bragg reflection. (*a*) An X-ray image at the peak of the crystal-integrated rocking curve. (*b*) A color map of the Bragg reflection angular widths (FWHM) and (*c*) a color map of the center of mass (COM) of the Bragg reflection angular dependences. All color maps presented in the paper are calculated with binning number *N* = 10. The contrast observed in (*a*) is mostly due to wavefront distortions in the beamline Be window (see Appendix *B*).

Fig. 1 ▸(*a*) presents an X-ray image of our reference silicon crystal in the 531 Bragg reflection recorded at the crystal-integrated Bragg reflection rocking curve maximum [displayed in Fig. 14(*a*) of Appendix *A* [App appa]]. Fig. 1 ▸(*b*) shows a color map of the Bragg reflection angular widths Δθ (full width at half-maximum, FWHM), while Fig. 1 ▸(*c*) shows a color map of the Bragg reflection peak relative angular positions θ, evaluated as a center of mass (COM) of the rocking curves. We note that the rippled background and the two straight lines in Figs. 1 ▸(*a*)–(*c*) are artifacts caused by beamline X-ray optical components (see Appendix *B* [App appb] for more details, and Section 6 for supplementary RCI measurements with improved beamline X-ray optics).

The RCI data provide access to numerous crystal parameters that are calculated by the RCI data evaluation code. The parameters used in these studies and their definitions are summarized in Table 1 ▸.

**Figure 2:**
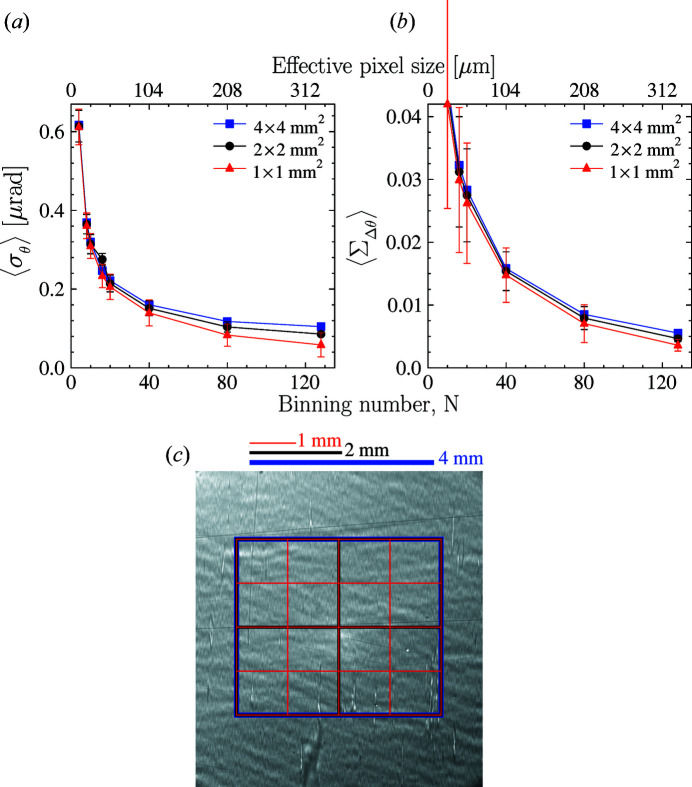
Averaged Bragg-plane slope errors 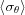 (*a*) and normalized dispersions 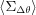 of the Bragg reflection widths (*b*) for the reference Si crystal in the 531 Bragg reflection measured and calculated as a function of the area-detector binning number *N*. The averaging is performed in one of three ways over 16 equal-sized 1 mm × 1 mm crystal’s ROIs, over four 2 mm × 2 mm ROIs, or just calculated in one 4 mm × 4 mm ROI, as indicated by red, black and blue lines, respectively, in (*a*) and (*b*) and on the X-ray Bragg diffraction image in (*c*).

Of these parameters, the Bragg-plane slope error 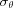 is one of the most important in the present studies. Fig. 2 ▸(*a*) shows 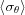 values in the reference silicon crystal as a function of the binning number *N* calculated in different regions of interest (ROIs) indicated by appropriate colors in Fig. 2 ▸(*c*). The error bars represent the variation in the 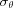 values being averaged. The data binning number *N* is related to the method of adding (binning) the signal from adjacent *N* × *N* area-detector pixels together to achieve a better signal-to-noise ratio or to minimize the effects of small observation errors at the cost of resolution.

The binning procedure moderates the impact of imperfections in the beamline X-ray optics, as discussed in more detail in Appendix *B* [App appb]. Remarkably, there is no significant change in the 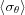 values measured in ROIs of different sizes, indicating the homogeneous quality of the reference silicon crystal and its fairly flat crystal planes over relatively large crystal areas. The average specific Bragg-plane slope errors are 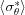 ≃ 0.1–0.05 µrad mm^−2^. These numbers may not necessarily represent the true value of the Bragg-plane slope errors in the silicon crystal. They might be even smaller (see supplementary data in Section 6). Rather, these numbers represent the resolution of our setup, limited by wavefront distortions in the beamline X-ray optical components.

RCI data also provide access to the dispersion 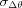 of the rocking curve widths Δθ. Normalized to the Bragg reflection width Δθ, this is a measure of the deviation from the largest Bragg reflectivity value for the given reflection. This can be easily understood, because the product of the Bragg reflection width and the reflectivity is an invariant value to a first approximation. Fig. 2 ▸(*b*) shows a plot of the normalized and averaged Bragg width dispersion 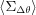 values (see Table 1 ▸ for the definition) in the reference silicon crystal as a function of the binning number calculated in ROIs of different sizes. Similar to the case of 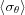 values in Fig. 2 ▸(*a*), there is no significant change in the 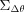 values with the size of the ROI. This is another indication of the very high and homogeneous quality of the reference silicon crystal. The specific average normalized Bragg width dispersion in silicon is 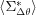 ≃ 0.003–0.005 mm^−2^. This is a small value, which indicates that the maximum Bragg reflectivity may be reduced by less than 0.5% due to crystal strain. Similar to the case of 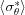, the small value of 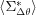 we measure in silicon may represent the resolution limit of the setup rather than the real value for single-crystal silicon, which may be even smaller (see supplementary data in Section 6).

## 3. Bragg-plane slope errors in diamond crystals   

**Figure 3:**
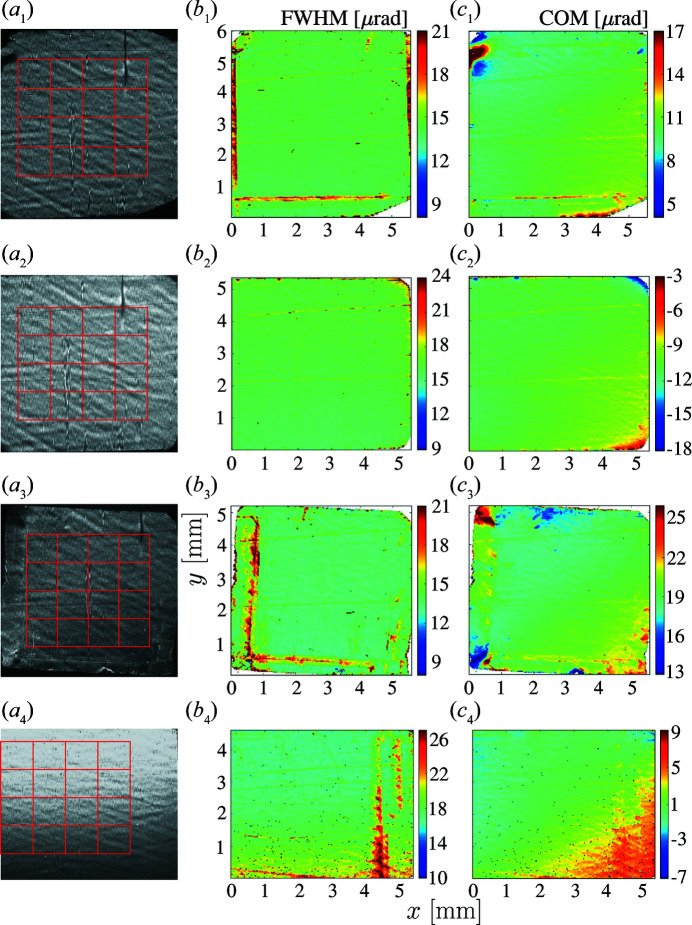
X-ray 400 Bragg reflection images and RCI maps for four selected type IIa HPHT diamond crystal plates in the (100) orientation. Columns are as in Fig. 1 ▸: (*a*) X-ray images at the peak of the crystal-integrated rocking curve, (*b*) the Bragg reflection angular widths and (*c*) the center of mass of the Bragg reflection angular dependences. Rows correspond to crystals: (1) VB4, (2) VB5, (3) VB6 and (4) D3. The red grids in column (*a*) indicate the ROIs, similar to Fig. 2 ▸(*c*).

A study of the Bragg-plane slope errors in diamond crystals was performed on samples selected using white-beam X-ray topography. All crystals are of type IIa grown by the HPHT technique, cut and polished to plates in the (100) orientation (Polyakov *et al.*, 2011 ▸). Two of the available crystals (termed VB4 and VB5) feature large areas (∼4 mm × 4 mm) free of dislocations, stacking faults, inclusions and other defects detectable by white-beam X-ray topography. Crystal VB4 was used previously in diamond Bragg reflectivity studies (Shvyd’ko *et al.*, 2011 ▸). X-ray Bragg diffraction images and RCI maps of these two crystals are shown in the upper two rows of Fig. 3 ▸. The RCI maps are homogeneous in the central region, revealing in particular a Bragg reflection width of Δθ = 14.8 µrad, close to the theoretical value. Another crystal (termed VB6) features a few weak dislocation lines in the central part. Stacking faults at the edges result in propagating strain fields, as well as areas with enlarged rocking curve widths, clearly seen on the RCI maps in row 3 of Fig. 3 ▸. X-ray white-beam topography reveals more defects and propagating strain fields in a crystal labeled D3. The relevant RCI data in row 4 reveal a crystal quality inferior to that of crystals VB4, VB5 and even VB6. All the crystal plates are rather thick: crystal VB4 is 1 mm, while the others are about 0.5 to 0.6 mm thick.

To avoid any externally induced strain, which could be caused for example by crystal mounting, the crystals lie free in a flat 1 mm-deep indentation machined in an aluminium block fastened to the stage used to perform angular scans. The indentation holding the diamond crystal in it was covered with a thin plastic foil to minimize the effect of air circulation on the angular stability of the crystal.

**Figure 4:**
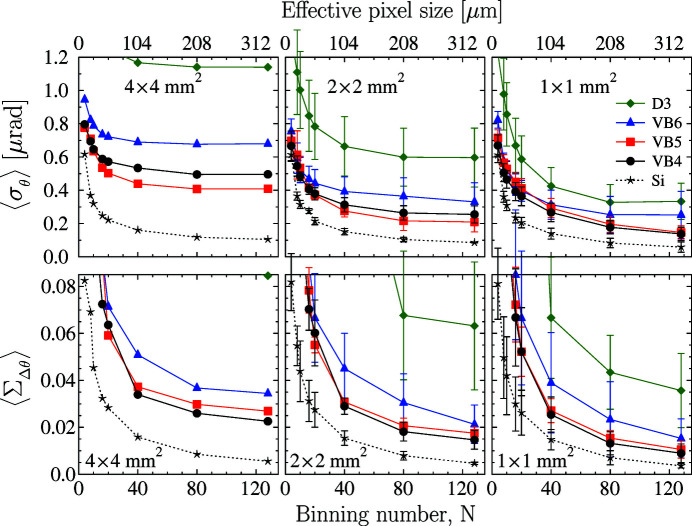
Averaged Bragg-plane slope errors 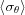 (upper row) and averaged and normalized Bragg-reflection width dispersions 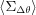 (lower row) in selected diamond crystals measured in the 400 Bragg reflection and calculated from the RCI data as a function of the area-detector binning number *N*. The data evaluation and averaging are performed in selected ROIs indicated by the red grids in Fig. 3 ▸.

The upper row of Fig. 4 ▸ shows the averaged Bragg-plane slope errors 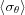 measured and calculated in ROIs of different sizes for the four selected freestanding crystals. Data for the reference silicon crystal are also presented for reference. The best crystal regions with the lowest densities of defects are selected for this purpose, as indicated by red grids in Figs. 3 ▸(*a*_1_)–3(*a*_4_). The smallest slope errors are observed as expected in crystals VB4 and VB5, in which the defects appear only on the crystal rims outside the selected ROIs. Unlike in silicon, the slope errors in these diamond crystals, even in the best crystals (VB4 and VB5), change substantially with the size of the ROI. This result indicates that the diamond crystal quality is less homogeneous than that of silicon.

Nevertheless, the averaged specific slope errors 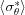 in the two best diamond crystals VB4 and VB5 (practically flawless in the central 4 mm × 4 mm ROI) feature values of 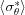 ≃ 0.15–0.2 µrad mm^−2^, only a factor of two larger than those in silicon. The overlapping error bars indicate that in some ROIs the specific errors in diamond and silicon are even comparable.

Similarly, the graphs in the lower row of Fig. 4 ▸ present plots of the averaged and normalized Bragg-reflection width dispersions 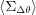 in the selected diamond crystals measured and calculated from the RCI data as a function of the area-detector binning number *N*. The 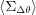 values calculated in ROIs of various sizes are quite different, thus revealing again, in agreement with the 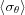 values, more inhomogeneities in the diamond crystals than in the reference silicon crystal. Nevertheless, the specific values presented in the 1 mm × 1 mm graph can be small, especially for the highest quality crystals (VB4 and VB5), approaching 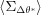 ≃ 0.01–0.013 mm^−2^, only a factor of two larger than the relevant reference silicon values.

These data indicate that the local reflectivity values in the two best diamond crystals are reduced (possibly by residual crystal strain) by no more than 1 to 1.3% from the maximum possible value. This result is in agreement with direct absolute reflectivity measurements previously performed on crystal VB4 (Shvyd’ko *et al.*, 2011 ▸). The peak reflectivity measured with an X-ray beam of cross section 1 mm × 1 mm and averaged over the central crystal area of 1 mm × 4 mm was 99.1 ± 0.4%, which is close to the theoretical value of 99.7%. We note that the specific 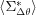 values presented here are evaluated on a larger crystal area of 4 mm × 4 mm. As discussed in Section 6, smaller 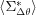 values, in even better agreement with the results of Shvyd’ko *et al.* (2011 ▸), can be measured with improved beamline X-ray optics.

## 4. Impact of diamond crystal clamping   

The data presented in the previous section were obtained on freestanding crystals. However, for optical components to function properly, they must be rigidly mounted in crystal holders to ensure angular and position stability. Correct mounting also provides for thermal transport to discharge the X-ray beam power absorbed by the crystal.

Even though the selected crystal plates are 0.5 to 1 mm thick and therefore very stiff due to the very large Young’s modulus of diamond, clamping without any precautions produces tremendous strain. A standard approach to reducing mounting strain is to introduce strain-relief cuts.

**Figure 5:**
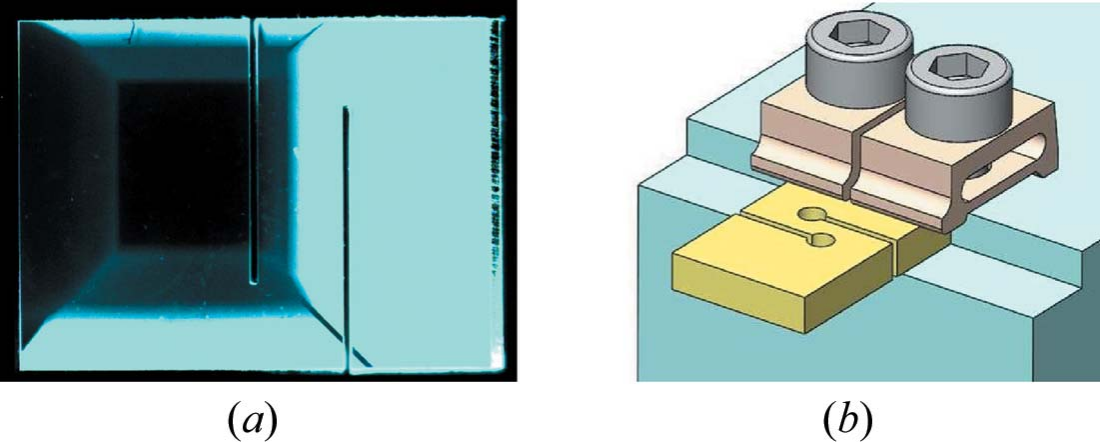
Specially designed and manufactured type IIa HPHT diamond crystal plate in the (100) orientation furnished with strain-relief cuts. (*a*) A UV-excited luminescence image. (*b*) A schematic diagram of the crystal with strain-relief features, clamped in a crystal holder.

A high-quality type IIa HPHT 440 µm-thick diamond crystal plate in the (100) orientation was selected, featuring a small number of defects only at the crystal rim. It was cut to a rectangular 5.4 mm × 4.5 mm plate and furnished with strain-relief cuts, as seen on the UV-excited luminescence image in Fig. 5 ▸. The darkest zone in the UV image corresponds to the (100) growth sector with the lowest nitrogen content. The strain-relief cuts are the two vertical parallel dark lines. The cuts were introduced to prevent the propagation of strain into the working area (on the left of the left cut) provided the crystal is clamped rigidly on the right of the right cut.

The clamping mechanism design is similar to that presented by Samoylova *et al.* (2019 ▸).

The cuts were made with YAG:Nd laser pulses in the second harmonic with duration 100 ns, energy 1.7 mJ per pulse, spot size 20 to 25 µm and repetition rate 5 kHz. The width of the cuts is ∼50 µm, made in two passes with a 25 µm lateral shift. Finite-element analysis shows that adding holes at the end of the cuts, as shown in Fig. 5 ▸(*b*), may produce better strain relief; however, such holes were not implemented for this particular sample.

**Figure 6:**
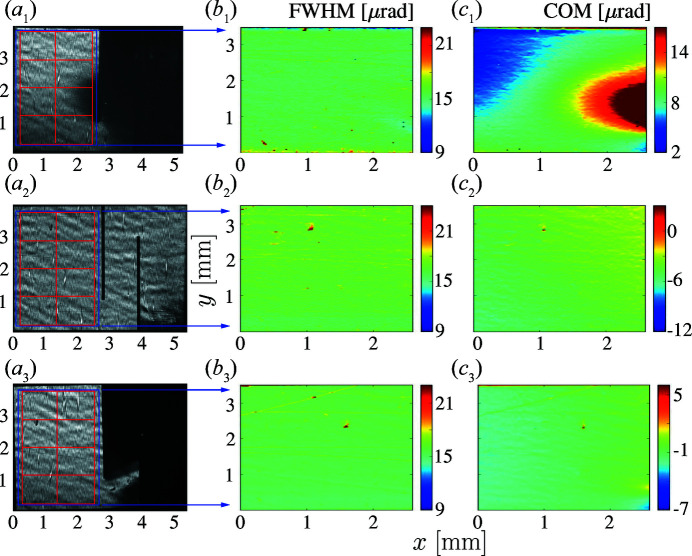
X-ray 400 Bragg reflection images and RCI maps for a mounted type IIa HPHT diamond crystal with strain-relief cuts. Columns are as in Fig. 3 ▸: (*a*) X-ray 400 Bragg reflection images at the peak of the crystal-integrated rocking curve, (*b*) Bragg reflection angular widths and (*c*) centers of mass of the Bragg reflection angular dependences in the ROIs indicated by the red grids in column (*a*). Rows correspond to the following conditions: (1) after laser cutting, (2) after annealing in air at 630°C for 3 h, (3) after clamping the right-hand part in the crystal holder.

Fig. 6 ▸(*a*_1_) shows an X-ray 400 Bragg diffraction image at the top of the crystal integrated rocking curve. The image reveals that the laser cutting induces a very large strain: only the working area reflects X-rays and can be imaged. The rest of the crystal is out of reflection because of the cutting-induced strain. The FWHM map in the working area shown in Fig. 6 ▸(*b*_1_) is very homogeneous, revealing an almost theoretical Bragg reflection width, which proves almost defect-free crystal quality. However, the COM map presented in Fig. 6 ▸(*c*_1_) reveals a very large strain, in agreement with Fig. 6 ▸(*a*_1_).

Is it possible to eliminate the strain induced by laser cutting? In our previous studies (Kolodziej *et al.*, 2016 ▸) we found that annealing diamond crystals in air at a temperature of 630–650°C for 3 h may substantially reduce strain induced in the process of laser cutting or ablation. The strain is caused by the graphitization of the machined surfaces. The annealing temperature is chosen such that all residuals of graphite and other carbon compounds are burned in air, while keeping the diamond intact^1^ We will refer to this procedure in the following as medium-temperature in-air annealing (MTA).

Indeed, such annealing practically erases the cutting-induced strain, as the X-ray Bragg diffraction image in Fig. 6 ▸(*a*_2_) and the RCI maps in Figs. 6 ▸(*b*_2_)–6 ▸(*c*_2_) evidence. These measurements were performed on a free-standing crystal in the configuration described previously.

The X-ray Bragg diffraction images and RCI maps in Figs. 6 ▸(*a*_3_)–6 ▸(*c*_3_) show what happens to the crystal if it is rigidly clamped, as presented schematically in Fig. 5 ▸(*b*). At first glance the image in Fig. 6 ▸(*a*_3_) resembles the case of Fig. 6 ▸(*a*_1_): only the working area can be imaged, while the rest is heavily strained and is out of reflection. In reality, the new situation is completely different. The COM map of the working area of the clamped crystal (on the left of the left cut) in Fig. 6 ▸(*c*_3_) looks very similar to the COM map of the freestanding annealed crystal in Fig. 6 ▸(*c*_2_). This demonstrates that clamping of a crystal furnished with strain-relief cuts does not produce strain in the working area.

**Figure 7:**
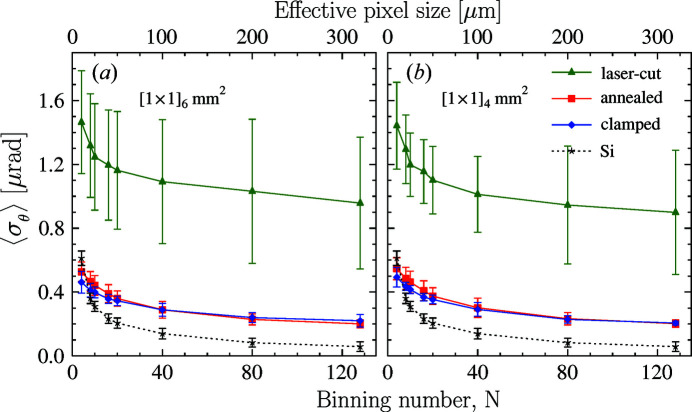
Averaged specific Bragg-plane slope errors 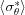 in the diamond crystal with strain-relief cuts after laser cutting (green), after MTA annealing (red) and after clamping (blue), evaluated as a function of the area-detector binning number *N* and compared with the reference silicon crystal values. The averaging is performed over either (*a*) the top six or (*b*) the top four of the eight equal-sized 1 mm × 1 mm ROIs indicated in Figs. 6 ▸(*a*_1_)–6 ▸(*a*_3_).

The averaged specific Bragg-plane slope error values 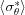 presented in Fig. 7 ▸ support this statement. Both graphs show 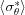 dependences on the binning number for the crystal after laser machining (green lines and markers), after MTA annealing (red) and clamped (blue). The averaging is performed either over (*a*) the top six or (*b*) the top four of eight equal-sized 1 mm × 1 mm ROIs indicated in Figs. 6 ▸(*a*_1_)–6 ▸(*a*_3_). The ROIs are indicated by red grids in Figs. 6 ▸(*a*_1_), 6 ▸(*b*_1_) and 6 ▸(*c*_1_), respectively. These data demonstrate that, first, annealing indeed helps to reduce the slope errors substantially to values of 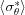 ≃ 0.2 µrad mm^−2^, which are very close to those observed for the best freestanding diamond crystals as documented in Fig. 4 ▸ (upper right graph). Second, and most important, the clamping does not degrade the observed slope errors in the upper working area. Extending averaging to all eight equal-sized 1 mm × 1 mm ROIs results in an increase in 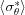 (data not shown), indicating that the working area should be limited to the top 2 mm × 2 mm or at most to the top 2 mm × 6 mm zone.

## 5. Effect of high-temperature annealing   

In the previous section it was shown that medium-temperature annealing of diamond crystals at ∼630–650°C in air helps to erase crystal strain induced by laser machining and improve slope errors to the baseline values.

Here we study the effect of annealing diamond crystals at higher temperatures. Vacancies, impurity atoms such as nitrogen and some other nanoscale crystal defects cannot be detected by X-ray topographies, but they may still contribute to the Bragg-plane slope errors. The mobility of vacancies, impurity atoms and other defects increases at higher temperatures in condensed matter systems (Cahn & Haasen, 1996 ▸). The expectations are that, in this process, the defects may be pushed to the crystal surfaces and growth zone boundaries where they annihilate and reduce strain. In diamond such processes start at about 900°C, but the highest temperature should be kept substantially lower than ∼2450°C, the Debye–Waller temperature of diamond.

In our experiments, we annealed diamond crystals at 1450°C for 3 h under high-vacuum conditions (∼4 × 10^−6^ mbar). We refer to this procedure as high-temperature high-vacuum (HTHV) annealing; Appendix *C* [App appc] gives technical details.

**Figure 8:**
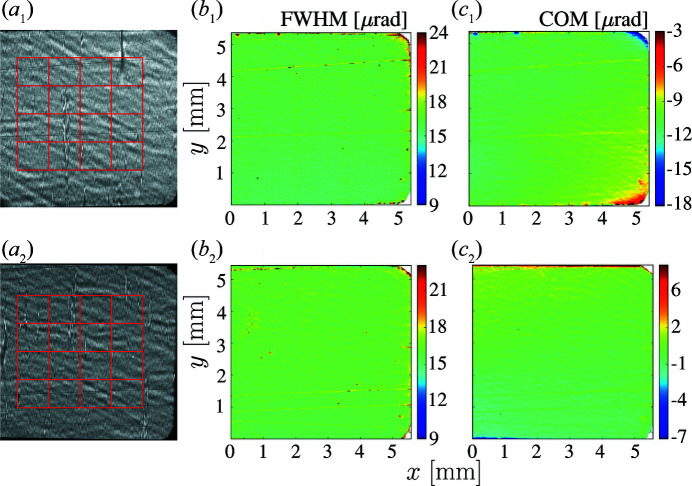
X-ray 400 Bragg reflection images and RCI maps for diamond crystal VB5 after annealing. Columns are as in Fig. 3 ▸: (*a*) X-ray images at the peak of the crystal-integrated rocking curve, (*b*) the Bragg reflection angular width and (*c*) the centers of mass of the Bragg reflection angular dependences. Rows correspond to (1) medium-temperature annealing in air (MTA) and (2) high-temperature high-vacuum annealing (HTHV).

Fig. 8 ▸ shows X-ray 400 Bragg diffraction images and RCI maps of diamond crystal VB5, one of the two best crystals used in this study. The top row shows the results after MTA but before HTHV annealing, while the bottom row presents data after HTHV annealing. The upper row is the same as row 2 in Fig. 3 ▸. There is a clear improvement in the homogeneity of the COM map, indicating also a reduction in the Bragg-plane slope errors 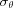.

**Figure 9:**
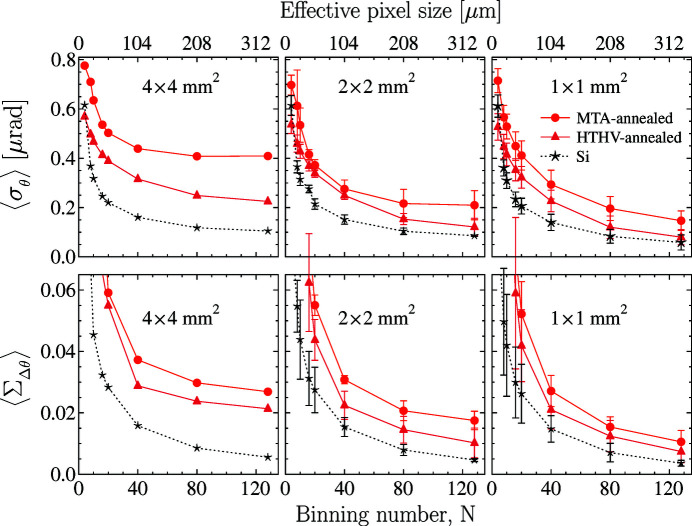
Similar to Fig. 4 ▸ (top), here showing averaged Bragg-plane slope errors 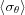 in the VB5 crystal before (red circles and curves, same as in Fig. 4 ▸) and after the HTHV annealing (brown triangles and curves), together with the reference silicon crystal data (black stars and curves).

Indeed, the 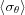 plots presented in Fig 9 ▸ support this assumption. Although there are still easily recognizable differences in the 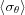 values calculated in ROIs of different sizes, the differences are not as large as before the HTHV annealing. The values after HTHV annealing approach the appropriate reference silicon values. Most striking, the averaged specific slope error values are reduced by almost a factor two to ∼0.1 µrad mm^−2^, becoming very close to the reference silicon value.

It is tempting at this point to draw a general conclusion that HTHV annealing substantially improves the Bragg-plane slope errors in diamond crystal and makes them close to those of silicon. This is most probably true if we are working with very high quality crystals like VB5. Unfortunately, this conclusion is not universally applicable. The HTHV annealing of diamond crystal VB6, which features some residual dis­location lines in the crystal center (in contrast to VB5, which is free of such defects), does not result in the same improvements as in the case of VB5. The very limited number of high-quality samples available for our studies does not allow us to make a universal conclusion. As stated before, it is most probable that HTHV annealing is efficient in improving Bragg-plane slope errors and pushing them to the silicon reference limit only in high-quality diamond crystals that have no dislocations, stacking faults and so forth in the ROI. Improvements are likely to be due to annihilation of vacancies, impurity atoms and so forth. These statements should be confirmed by studies of a larger set of high-quality diamond crystals.

## 6. Bragg-plane slope errors remeasured after replacing beamline Be window with a polished one   

In the present study, we have applied the binning procedure to moderate the detrimental impact of wavefront distortions due to imperfections in beamline X-ray optics on the true values of the Bragg-plane slope errors 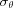 and normalized dispersions 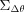 of the Bragg reflection widths (see Appendix *B* [App appb]). A better approach would be instead to improve the beamline optics, most importantly the beamline Be window (see Fig. 13), the main source of the distortions. Indeed, the window was replaced with a new polished one, but unfortunately after the paper was submitted for publication. Luckily, before the paper was accepted for publication, we were able to perform supplementary measurements with the new Be window and compare them with the results obtained with the old un­polished window. We cannot redo all the measurements, because the diamond crystals were modified irreversibly by annealing and other procedures. In this section we provide results of a comparative study of the 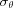 and 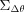 values measured with the old and the new Be windows in the reference Si crystal (see Fig. 2 ▸ for original data) and in diamond crystal sample VB5, featuring the smallest 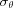 and 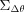 values obtained after HTHV annealing (see Fig. 9 ▸ for original data).

**Figure 10:**
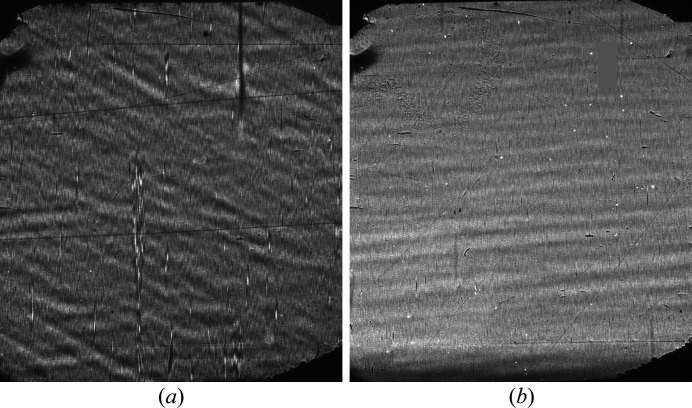
X-ray images of diamond crystal VB4 in the 400 Bragg diffraction taken at the peak of the rocking curve with (*a*) the old and (*b*) the new polished beamline Be windows.

Fig. 10 ▸ shows, as examples, X-ray images of one of the diamond crystal samples measured with (*a*) the old and (*b*) the new Be windows. These are the type of images shown in Fig. 3 ▸. High-contrast vertical streaks and high-contrast wavy features on the image in Fig. 10 ▸(*a*) taken with the old window dis­appear from the image in Fig. 10 ▸(*b*) taken with the new window. Wavy features are still present in Fig. 10 ▸(*b*), but with a much lower contrast. As a result, diamond crystal imperfections can be now better identified and separated from beamline X-ray optics imperfections.

**Figure 11:**
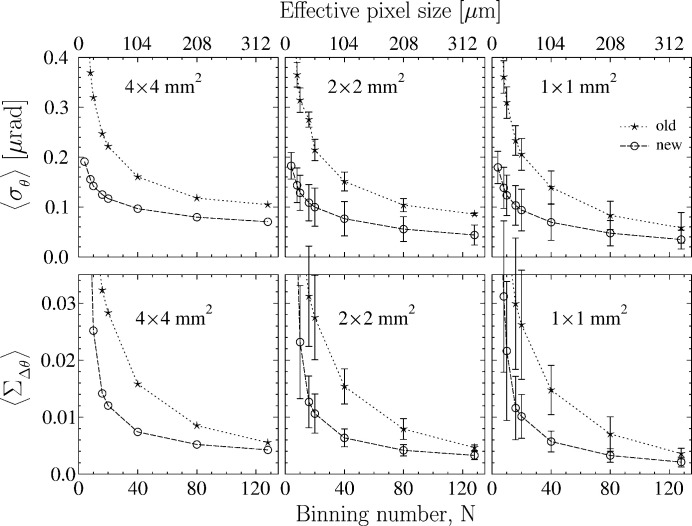
A comparison of averaged Bragg-plane slope errors 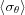 (upper row) and normalized dispersions 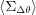 of the Bragg reflection widths (bottom) for the reference Si crystal in the 531 Bragg reflection measured with the old unpolished (stars) and the new polished (open circles) Be windows. The 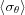 and 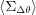 values are calculated as a function of the area-detector binning number *N* in 4 mm × 4 mm, 2 mm × 2 mm or 1 mm × 1 mm ROIs, similar to Fig. 2 ▸.

**Figure 13:**
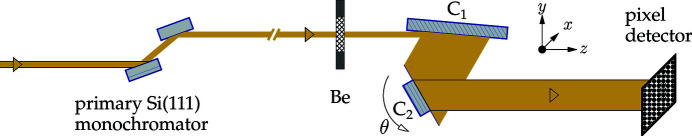
The layout and optical components of the rocking curve imaging setup at the Advanced Photon Source (APS) on bending magnet beamline 1BM, comprising a primary double-crystal Si(111) monochromator, beryllium (Be) window, conditioning crystal C_1_, crystal under study C_2_ and pixel detector.

**Figure 14:**
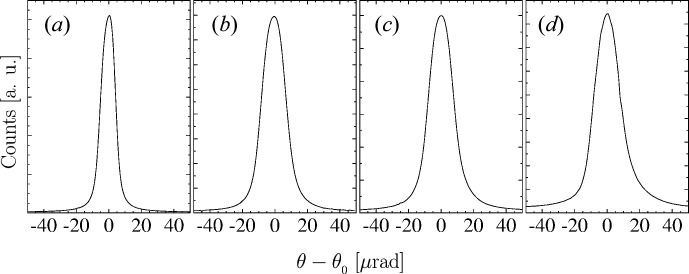
The crystal-integrated angular dependences of Bragg reflectivity (rocking curves) of the crystals used in these experiments: (*a*) the reference silicon crystal in the 531 Bragg reflection, and diamond crystals (*b*) VB5, (*c*) VB6 and (*d*) D3 in the 400 Bragg reflection. The Bragg reflection widths (FWHMs) are (*a*) 10.3 µrad, (*b*) 17.0 µrad, (*c*) 17.5 µrad and (*d*) 18.7 µrad. The measurements were performed in the double-crystal C_1_–C_2_ arrangement shown schematically in Fig. 13 ▸ with an Si PIN diode used instead of the pixel detector. See text for more details.

The positive impact of the new polished Be window on the 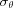 and 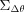 values in the reference Si crystal and diamond crystal VB5 are clearly seen from the data presented in Figs. 11 ▸ and 12 ▸, respectively. The binning-number dependencies 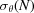 in selected ROIs are presented in the top rows of the figures, while the 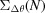 dependencies are shown in the bottom rows.

We observe three effects. First, all the 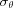 and 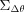 values become smaller with the new Be window. The 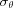 values evaluated with smallest binning numbers *N* experience the largest reduction by almost a factor of three. Second, all dependences related to the new window approach asymptotic values more rapidly at large *N*. Third, the data points measured with both mirrors converge at large *N* values.

The new measurements support our prediction that the binning-number dependences would become flat if perfect optics were used. The comparative studies allow us to draw the conclusion that the 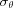 and 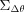 values evaluated at large binning numbers represent the upper limit of the true crystal values. These values are tabulated in Table 2 ▸.

In particular, Table 2 ▸ shows averaged specific Bragg-plane slope errors 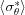 and averaged normalized dispersions 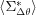 of the Bragg reflection widths in the reference silicon crystal and in the best diamond crystal VB5 (after HTHV annealing) calculated with binning number 80 from data measured with the old and the new beamline Be windows. The numbers in brackets represent the last-digit variation range for the provided numbers over 16 selected 1 mm × 1 mm crystal ROIs. They show that the 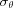 and 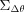 values in particular ROIs of diamond can approach those measured in the reference silicon crystal.

## 7. Conclusions and outlook   

Perfect crystals with flat Bragg planes are a prerequisite for wavefront preservation in Bragg diffraction. We have used an X-ray rocking curve imaging (RCI) technique to study the smallest achievable Bragg-plane slope errors in the best available synthetic diamond crystals and how they compare with those in the highest quality reference silicon crystals.

We have shown that the smallest specific slope errors in the best diamond crystals are about 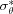 ≃ 0.08 (3) µrad mm^−2^, which are only 50% larger than the ∼0.05 (2) µrad mm^−2^ slope errors we measured in the reference silicon crystals. In particular 1 mm × 1 mm ROIs of the best diamond crystal, the 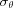 values can approach those measured in the reference silicon crystal.

RCI data also provide access to the normalized specific dispersion 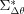 of the rocking curve widths Δθ, which is a measure of the deviation from the largest Bragg reflectivity achievable by a perfect crystal. The best diamond crystals feature normalized specific dispersion values 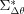 ≃ 0.006 (2) mm^−2^ *versus* ∼0.003 (1) mm^−2^ in silicon. These data indicate that the local reflectivity values in the best diamond crystals are reduced by not more than 1% from the maximum values, in agreement with previous Bragg reflectivity studies in diamond (Shvyd’ko *et al.*, 2011 ▸).

The small slope errors are achieved not only in freestanding diamond crystals but also in crystals firmly mounted in crystal holders, provided the crystals are designed and machined with special strain-relief features.

High-temperature annealing of the best diamond crystals at 1450°C reduces the Bragg-plane slope errors to values approaching those of silicon.

Further investigations are in progress to establish the wavefront-preservation properties of the best available diamond crystals.

## Figures and Tables

**Figure 12:**
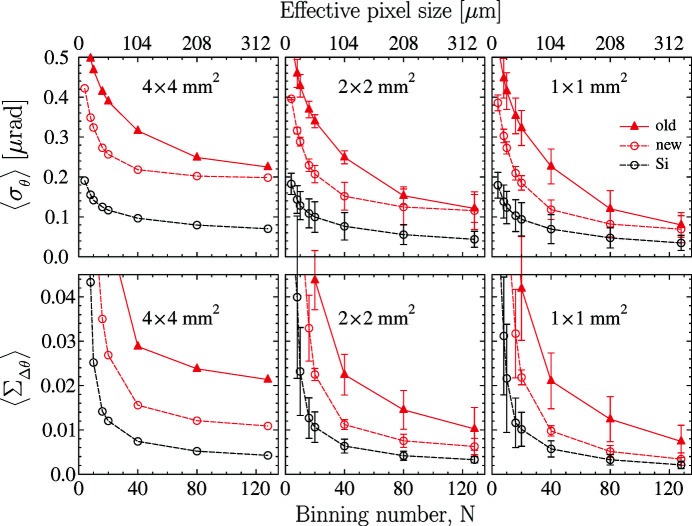
Similar to Fig. 11 ▸, showing here 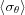 values in the upper row and 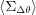 values in the bottom row for HTHV annealed diamond crystal VB5 in the 400 Bragg reflection measured with the old (red triangles) and the new (red open circles) beamline Be windows. Open black circles show the results for the reference Si crystal measured with the new Be window.

**Figure 15:**
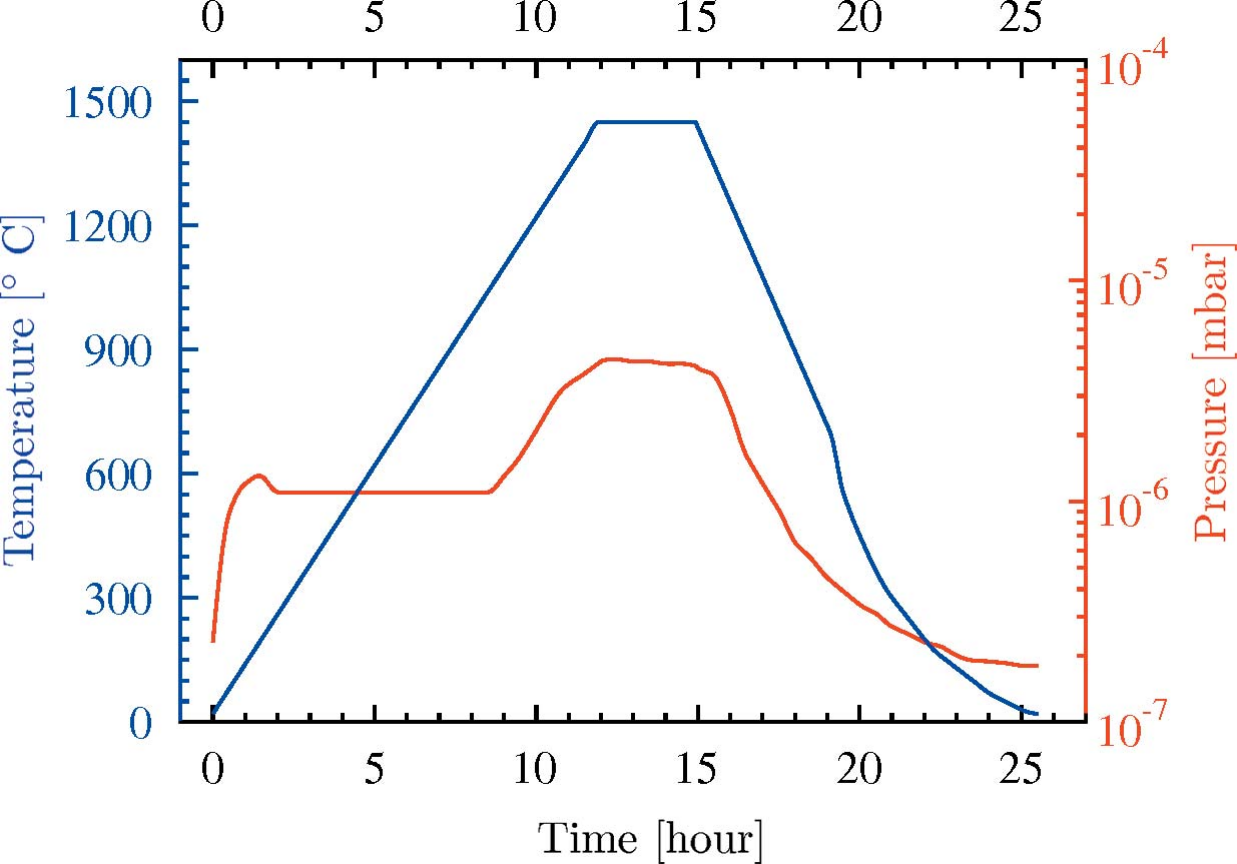
The time-dependence of temperature and pressure in the high-temperature high-vacuum furnace during simultaneous annealing of diamond crystals VB5 and VB6.

**Table 1:** Notations and definitions of the characteristic crystal values measured and evaluated with the X-ray rocking curve imaging (RCI) technique

Notation	Definition
θ	Relative angular position of the Bragg reflection peak (center of mass, COM) measured at a particular location on the crystal by an area-detector pixel
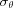	Bragg-plane slope error calculated as the dispersion of the θ values within a selected region of interest (ROI) on the crystal
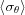	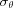 values averaged over multiple equal-sized ROIs
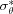 , 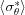	Specific 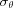 or 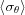 values measured over 1 mm × 1 mm ROIs

Δθ	Angular width (full width at half maximum, FWHM) of the Bragg reflection peak measured locally on the crystal by an area-detector pixel
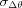	Dispersion of the Δθ values within an ROI
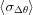	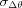 values averaged over several equal-sized ROIs
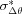 , 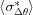	Specific 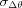 or 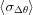 values measured over 1 mm × 1 mm ROIs
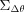 = 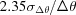 , 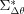 = 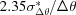 , *etc.*	Normalized 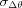 , 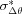 , 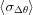 or 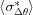 values

*N*	Data binning number, related to the method of adding (binning) the signal from adjacent *N* × *N* area-detector pixels together to achieve a better signal-to-noise ratio or to minimize the effects of small observation errors, at the cost of resolution

**Table 2:** Averaged specific Bragg-plane slope errors 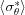 and averaged normalized dispersions 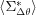 of the Bragg reflection widths in the reference silicon crystal and in the best diamond crystal VB5 (after HTHV annealing) calculated with binning number 80 from data measured with the old and the new beamline Be windows

	Old Be window		New Be window	
Crystal	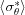	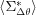	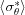	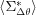
(reflection)	(µrad mm^−2^)	(mm^−2^)	(µrad mm^−2^)	(mm^−2^)
Si(531)	0.08 (3)	0.007 (3)	0.05 (2)	0.003 (1)
C*(400)	0.12 (5)	0.013 (5)	0.08 (3)	0.006 (2)

## Footnotes

1 Increasing the annealing temperature or time may result in etching of the crystal surface.

## Appendix A Rocking curve imaging

Bragg-plane slope errors in diamond and silicon crystals are measured using X-ray Bragg diffraction rocking curve imaging (RCI), also known as sequential topography (Lübbert *et al.*, 2000 ▸).

In this technique, the crystal under study is the second crystal (crystal C_2_ in Fig. 13 ▸) in a two-crystal nondispersive (or close to nondispersive) Bragg diffraction arrangement. The first (conditioning) crystal C_1_ is in a strongly asymmetric Bragg reflection geometry, with the asymmetry factor *b* chosen such that X-rays of each photon energy have a small angular spread  =  upon Bragg reflection [here  is the angular width of the symmetric reflection], much smaller than the Bragg reflection angular width of the second crystal, . Such asymmetric reflection from the conditioning crystal also results in an increased cross section of the reflected beam by a factor of |*b*|, capable of illuminating the whole of crystal C_2_ or at least the larger part of it. If the crystals are perfect, the Bragg reflection angular dependence (rocking curve) is very close to the intrinsic Bragg reflection angular dependence of the second crystal under study, C_2_. The conditioning crystal is typically manufactured from almost-perfect silicon single-crystal material.

A pixel area X-ray detector is used to measure X-ray Bragg reflection images sequentially at different incidence angles of X-rays to the Bragg-reflecting atomic planes of the second crystal. Figs. 1 ▸(*a*) and 3 ▸(*a*) show examples of such images measured in silicon and diamond crystals, respectively. They were recorded at the crystal-integrated Bragg reflection rocking curve peaks. Examples of crystal-integrated rocking curves are shown in Fig. 14 ▸. The RCI procedure can be seen as measuring rocking curves at particular locations of the second crystal with the area-detector pixels. The rocking curves measured with each detector pixel are used to calculate Bragg reflection maps. Figs. 1 ▸(*b*) and 3 ▸(*b*) show examples of the color maps of the Bragg reflection angular widths Δθ (full width at half-maximum, FWHM). Figs. 1 ▸(*c*) and 3 ▸(*c*) show examples of the color maps of the Bragg reflection peak relative angular positions θ, evaluated as a center of mass (COM) of the rocking curves. The RCI maps are calculated using a dedicated code (Stoupin, 2015 ▸), although this code does not correct for residual dispersion effects. The microscopic structure defects can be derived from the Bragg reflection FWHM maps. The mesoscopic and macroscopic crystal strain and Bragg plane slope errors can be best evaluated from the COM maps.

We use an X-ray diffraction sequential topography setup (Stoupin *et al.*, 2016*b* ▸) on the X-ray optics testing beamline 1BM (Macrander *et al.*, 2016 ▸) at the Advanced Photon Source (APS). The setup enables rocking curve mapping with a 25 nrad angular and 2.6 µm spatial resolution. The former is achieved using a high-precision KTG-15D goniometer (Kohzu Precision Co. Ltd) for crystal C_2_, while the latter is defined by the 6.5 µm pixel size of the ANDOR Neo-5.5 sCMOS camera and the ×2.5 magnification of the collecting light optic.

We study in this paper diamond crystal plates in the (100) orientation using the 400 Bragg reflection of 8 keV X-rays with Bragg angle  = 60.345°. The theoretically expected Bragg reflection width is  = 14.7 µrad (FWHM). Bragg reflection 531 from silicon with a Bragg angle  = 57.6° for 8 keV X-rays ( =  = 9.8 µrad) is the best match for the closest-to-nondispersive double-crystal setting with the 400 Bragg reflection from diamond. This reflection is used with the conditioning silicon crystal in the present study. The crystal is cut asymmetrically with asymmetry angle η = 55.6°, resulting in asymmetry parameter *b* =  ≃ −1/26. The angular spread of monochromatic X-rays upon such Bragg reflection is  =  = 1.9 µrad, which is much smaller than .

The *x* and *y* axes in Figs. 1 ▸(*a*)–1 ▸(*c*) and 4 ▸(*a*)–4 ▸(*c*), as well as in other X-ray Bragg diffraction images and RCI maps, correspond to the detector coordinates. The diffraction plane is parallel to the *y* axis. The X-ray Bragg diffraction images and RCI maps therefore appear to be contracted in the *y* direction by a factor of  ≃  ≃ 0.87.

The examples presented in Fig. 1 ▸ are based on measurements with a silicon crystal in the symmetric 531 Bragg reflection as the second crystal under study. Because the Bragg reflections from both crystals are the same in this case, the crystals are in a perfect nondispersive setting, with the rocking curves unaffected by the energy and angular spread of the X-rays, predominantly determined by the Bragg reflection parameters of the second crystal. Both silicon crystals (conditioning and reference) were manufactured from highest quality (high resistivity) almost-perfect single-crystal material.

Measurements with a silicon C_2_ crystal were performed to establish a reference for the diamond crystals and to benchmark the ultimate performance of the RCI setup used in this study. They are addressed in more detail in Section 2.

## Appendix B Moderating the impact of beamline wavefront distortions

As X-rays propagating from the bending-magnet source travel to the crystal under study they interact with numerous beamline optical components, including Bragg-reflecting silicon crystals in the primary high-heat-load monochromator, two successive beryllium windows and the conditioning crystal (see Fig. 13 ▸). Unfortunately, these interactions – primarily with the Be windows – introduce significant wavefront distortions. Most of the measurements presented in this paper (except those shown in Section 6) were made using a 25-year old compound Be window composed of two unpolished 0.25 mm-thick Be disks. The contrast observed on the X-ray Bragg diffraction images of the reference silicon crystal in Fig. 1 ▸(*a*) and the similar images of diamond crystals in Figs. 3 ▸(*a*_1_)–3 ▸(*a*_4_), such as the vertical streaks, rippled background and other irregular solitary features, are due to wavefront distortions and have nothing to do with crystal imperfections. These distortions result in local fluctuations in the direction and angular distribution of the X-rays incident on the crystal under study and therefore may perturb the local values of the Bragg reflection widths Δθ and peak positions θ, and thus falsify genuine RCI maps and RCI characteristics. Indeed, the RCI maps in Figs. 1 ▸(*b*) and 1 ▸(*c*) inherit the rippled background structure of the X-ray Bragg diffraction image in Fig. 1 ▸(*a*). This of course affects the calculated RCI characteristic values, such as and , and may lead to false conclusions regarding crystal properties.

The distortions observed in Fig. 1 ▸(*a*) have a characteristic length from ∼10 to ∼100 µm. The characteristic lengths of the Bragg-plane slope variations in the defect-free regions are typically larger. Therefore, the negative impact of the wavefront distortions on the RCI maps and on the characteristic values can be mitigated by smoothing these distortions. A standard procedure in such cases is adding together (binning) the signal recorded by *N* × *N* adjacent area-detector pixels to achieve a better signal-to-noise ratio and minimize the effects of observation errors, albeit at the cost of resolution.

Figs. 2 ▸(*a*) and 2 ▸(*b*) show plots of RCI characteristic values  and , respectively, as a function of the binning number *N* calculated in various crystal ROIs. The blue markers correspond to a 4 mm × 4 mm crystal ROI. The black markers correspond to averaged RCI values calculated in four equal-sized 2 mm × 2 mm ROIs, while the red markers correspond to averaged RCI values calculated in sixteen equal-sized 1 mm × 1 mm ROIs. The error bars indicate the scattering range of the values we are averaging. The particular ROIs are indicated by appropriate colors in Fig. 2 ▸(*c*).

The RCI characteristic values  and  of the reference silicon crystal first decrease rapidly with *N* and then reach steady values. If the beamline X-ray optics were perfect, we would have measured flat curves. The binning procedure minimizes the detrimental effect of the wavefront distortions due to the beamline optics. The genuine RCI characteristic values  and  of the reference silicon are close to those obtained with large binning numbers. This assumption is supported by supplementary measurements performed with a new 0.5 mm-thick polished mirror, presented in Section 6.

The binning procedure can ‘improve’ the RCI characteristic values  and  only if the wavefront contrast in X-ray Bragg diffraction images is larger than the contrast due to crystal defects. If a crystal is damaged or badly strained the binning procedure cannot help. Figs. 6 ▸(*a*_1_)–6 ▸(*c*_1_) show examples of X-ray Bragg diffraction images and RCI maps of a diamond crystal significantly strained by laser machining. In this case binning cannot ‘improve’ substantially the specific Bragg-plane slope errors , shown by green markers and lines in Fig. 7 ▸. The improvement in Bragg-plane slope errors occurs due to crystal annealing, as the other data in Fig. 7 ▸ demonstrate.

## Appendix C High-temperature high-vacuum annealing

A Red Devil G vacuum furnace manufactured by R. D. Webb Company Inc. was used for high-temperature high-vacuum annealing. Fig. 15 ▸ shows typical temperature and pressure profiles in the furnace during the annealing process. The heating rate was 2°C min^−1^ and the cooling rate was −3°C min^−1^. From 700°C the heating was switched off and the furnace was allowed to cool naturally.

The vacuum is ∼10^−7^ mbar at the beginning at room temperature, but it increases to ∼4 × 10^−6^ mbar at 1450°C. It is very important to have high-vacuum conditions to avoid diamond damage. However, even under such high-vacuum conditions the color of the diamond changes to light gray. The subsequent medium-temperature annealing in air erases the gray color and makes the diamond transparent again, suggesting that the color change is a surface rather than a bulk effect.
